# Perioperative Hyperoxia and Early Pulmonary Epithelial and Glycocalyx-Related Biomarker Trajectories in Laparoscopic Surgery: A Prospective Randomized Study †

**DOI:** 10.3390/life16071160

**Published:** 2026-07-14

**Authors:** Sevda Guliyeva, Mert Canbaz, Kübra Vardar, Nükhet Sivrikoz, Özlem Turhan, Zerrin Sungur, Uğur Aksu, Mert Şentürk

**Affiliations:** 1 Department of Anesthesiology and Reanimation, Istanbul Faculty of Medicine, Istanbul University, Istanbul 34093, Turkey; dr.sg257@gmail.com (S.G.); mcanbaz@ku.edu.tr (M.C.); ntsz06@gmail.com (N.S.); ozlemturhan6@gmail.com (Ö.T.); zsungur@istanbul.edu.tr (Z.S.); 2 Biology Department, Science Faculty, Istanbul University, Istanbul 34116, Turkeyuguraksu@istanbul.edu.tr (U.A.); 3 Department of Anesthesiology and Reanimation, School of Medicine, Acibadem University, Istanbul 34752, Turkey

**Keywords:** hyperoxia, normoxia, perioperative oxygen therapy, surfactant protein-A, syndecan-1, sialic acid, TNF-α, endothelial glycocalyx

## Abstract

Although perioperative oxygen therapy is a routine component of general anesthesia, its early biological consequences remain incompletely understood. This prospective randomized study evaluated whether perioperative oxygen concentration influences early biomarker responses in adults undergoing elective laparoscopic lower abdominal surgery. Patients received either normoxia (FiO_2_ 0.35) or hyperoxia (FiO_2_ 0.80) under standardized anesthesia. Clear physiological separation between groups was confirmed by arterial blood gas analysis. The primary biomarker finding was that circulating surfactant protein-A (SP-A) increased significantly in the normoxia group, whereas no comparable increase was observed under hyperoxia. Syndecan-1 and sialic acid showed descriptively similar directional patterns; however, these secondary biomarker findings were interpreted as exploratory and were not robust after Holm correction. By contrast, tumor necrosis factor-alpha (TNF-α) levels were higher postoperatively in the hyperoxia group, while ischemia-modified albumin (IMA) and total protein did not differ significantly between groups. These findings suggest that perioperative hyperoxia was associated with different early circulating biomarker trajectories across pulmonary epithelial and glycocalyx-related domains, without establishing pulmonary or endothelial protection. Further studies are needed to determine whether these early mechanistic findings translate into clinically meaningful outcomes.

## 1. Introduction

Perioperative oxygen therapy remains widely used during general anesthesia, yet its biological and clinical consequences remain unresolved. Higher inspired oxygen fractions have long been used not only to prevent hypoxemia but also with the expectation of reducing surgical site infections (SSI), but the overall evidence remains inconsistent [1,2]. In the PROXI trial, 80% oxygen did not reduce SSI or pulmonary complications after abdominal surgery, while long-term follow-up raised concern about increased mortality, particularly in patients undergoing cancer surgery [3,4]. More recent systematic reviews and meta-analyses have likewise emphasized that the benefits of liberal perioperative oxygen therapy remain uncertain and may be offset by harm in selected populations [2]. This uncertainty is also reflected in international guidance, as the WHO currently suggests—rather than strongly recommends—80% FiO_2_ for intubated adult surgical patients to reduce SSI [5].

This unresolved clinical picture is biologically plausible. Although supplemental oxygen is essential to avoid hypoxemia, supraphysiological oxygen exposure has been shown to influence redox balance, vascular tone, and inflammatory signaling [6,7]. Hyperoxia has also been associated with changes in oxidative stress biomarkers and antioxidant defenses in perioperative and critical care settings [8,9]. At the same time, perioperative molecular data are not uniformly adverse, and selected human studies have reported lower postoperative inflammatory responses under higher inspired oxygen fractions in specific surgical settings [10]. Yet most perioperative oxygen studies have focused primarily on clinical outcomes, whereas the early biological responses to oxygen exposure remain less clearly defined [2]. This is particularly relevant in laparoscopic surgery, where mechanical ventilation, pneumoperitoneum, and surgical stress may simultaneously influence pulmonary epithelial integrity and endothelial homeostasis.

Among the candidate biological domains, pulmonary epithelial response may be especially informative. Surfactant protein-A (SP-A), a surfactant-associated glycoprotein produced mainly by alveolar type II epithelial cells, is closely linked to alveolar epithelial stress and surfactant-related activity [11]. We also sought to determine whether any pulmonary signal was accompanied by parallel changes in markers of endothelial glycocalyx-related injury, since the endothelial glycocalyx is a dynamic surface layer that contributes to vascular permeability control, endothelial–blood cell interaction, mechanotransduction, and microcirculatory homeostasis; accordingly, syndecan-1 and sialic acid were included as complementary glycocalyx-related biomarkers [12,13,14,15]. To provide broader mechanistic context, ischemia-modified albumin (IMA) was assessed as a marker of oxidative and ischemia-related protein modification, reflecting a complementary aspect of redox-related disturbance, while tumor necrosis factor-alpha (TNF-α) was evaluated as a complementary marker of inflammatory activation, given its established role in perioperative and endothelial inflammatory signaling [16,17].

In this prospective randomized controlled trial, we examined the impact of perioperative oxygen concentration on circulating biomarkers in adults undergoing elective laparoscopic lower abdominal surgery under standardized general anesthesia. Patients were assigned to either a normoxic strategy (FiO_2_ = 0.35) or a hyperoxic strategy (FiO_2_ = 0.80). The primary outcome was the perioperative SP-A response to oxygen strategy, defined as the between-group difference in change from T0 to T1 and assessed by the group × time interaction. Secondary biomarker outcomes included syndecan-1, sialic acid, IMA, TNF-α, and total protein, which were treated as secondary exploratory biomarker outcomes. We hypothesized that perioperative oxygen exposure would be associated with distinct early SP-A responses, together with differential changes across complementary glycocalyx-related, oxidative, and inflammatory biomarker domains in an exploratory mechanistic setting.

## 2. Materials and Methods

### 2.1. Study Design and Ethical Approval

This investigation was structured as a prospective, randomized, single-center trial conducted within the Anesthesiology and Reanimation clinic at the Istanbul Faculty of Medicine, Istanbul University. Before initiating any study-related procedures, formal ethical endorsement was granted by the local Clinical Research Ethics Board (Reference: 2017/771, approved on 23 June 2017; URESIN). All enrolled individuals provided their explicit written consent. The entire research process was governed by the ethical mandates of the Declaration of Helsinki, while the final manuscript drafting followed the CONSORT framework. The study was retrospectively logged in the ClinicalTrials.gov registry (Registration No: NCT07525661) after patient enrollment had already begun. Additional chronology, endpoint-prespecification, and document-consistency details are provided in Supplementary File S1.

### 2.2. Participants

Individuals considered for enrollment were adults spanning 18 to 70 years of age, designated for elective lower abdominal laparoscopic procedures. Inclusion required an ASA physical status score between I and III, a BMI spanning 18.5 to 35 kg/m^2^, and an expected operative time of more than 1.5 h managed via a standardized volume-controlled general anesthetic approach. Criteria for exclusion involved baseline room-air oxygen saturation under 92%, chronic respiratory diseases requiring ongoing medication, severe heart disease, marked hepatic or renal impairment, systemic infections, and the concurrent use of steroid or immunosuppressive therapies. We additionally excluded pregnant women, individuals anticipating postoperative ventilatory support, subjects with known airway difficulties, and those lacking the capacity to consent.

### 2.3. Randomization and Allocation

Participants were assigned in a 1:1 ratio to the normoxia or hyperoxia arm using a computer-generated randomization list prepared before recruitment by an investigator with no role in intraoperative care or data collection. Group concealment was maintained with sequentially numbered, opaque, sealed envelopes, which were opened after induction of anesthesia. Because of the nature of the intervention, the anesthesia team could not be blinded; however, laboratory staff and statisticians remained unaware of treatment allocation. The primary analysis followed a per-protocol framework because the study aimed to assess biomarker responses under protocol-defined oxygen exposure and ventilation conditions. Patients were therefore excluded from the primary analysis when biomarker samples were non-evaluable or when ventilation no longer followed the protocol-defined volume-controlled strategy. These exclusions were applied because valid assessment of the prespecified biomarker outcomes required both interpretable laboratory measurements and protocol-concordant exposure conditions.

### 2.4. Anesthesia and Ventilation Protocol

A uniform intraoperative protocol was followed for the administration of general anesthesia. Prior to anesthesia induction, patients were monitored using standard continuous electrocardiography, non-invasive blood pressure tracking, and pulse oximetry. Following induction, this was supplemented with end-tidal carbon dioxide (EtCO_2_) and invasive arterial blood pressure monitoring. Standard preoxygenation (FiO_2_ = 1.0) was performed on all participants before induction and kept constant until the trachea was intubated. Anesthesia was induced using intravenous administration of propofol (2–2.5 mg/kg), fentanyl (2 µg/kg), and midazolam (0.05 mg/kg). Endotracheal intubation via a cuffed tube was facilitated by rocuronium (0.6 mg/kg). Subsequently, mechanical ventilation was initiated in volume-controlled ventilation (VCV) mode via a LEON anesthesia workstation/ventilator (Löwenstein Medical SE & Co. KG, Bad Ems, Germany). Settings included a positive end-expiratory pressure (PEEP) of 5 cmH_2_O, an inspiratory-to-expiratory ratio of 1:2, and a tidal volume based on 7 mL/kg of predicted body weight. The respiratory rate was continuously modified to maintain EtCO_2_ levels within the 35–45 mmHg range. Post-intubation, participants were assigned to their respective oxygenation groups (either FiO_2_ 0.35 or 0.80). This assigned strategy was strictly maintained for the duration of the surgical procedure without any crossover. Thus, the randomized FiO_2_ strategy began immediately after intubation and after group allocation had already been determined. Oxygen was titrated using an air-oxygen blend at a constant total fresh gas flow of 2 L/min via the anesthesia workstation. For anesthesia maintenance, sevoflurane was combined with a continuous intravenous infusion of remifentanil (0.05–0.2 µg/kg/min), with dosages adjusted based on the patient’s hemodynamic needs. Standard surgical protocols were followed for the establishment and maintenance of pneumoperitoneum, typically reaching an intra-abdominal pressure between 10 and 12 mmHg. These pressure levels were dictated entirely by the surgical team and were not modified for the research. Furthermore, the management of intravenous fluids and vasoactive agents adhered to standard institutional guidelines tailored to each patient’s specific hemodynamic goals.

### 2.5. Arterial and Venous Blood Gas Analysis

Blood gas samples (both arterial and venous) were collected 10 min following the stabilization of the targeted inspired oxygen fraction. Peripheral venous catheters were used to draw venous blood, while arterial samples were acquired via an indwelling radial arterial catheter. The analysis included parameters such as pH, lactate levels, arterial and venous tensions of oxygen (PaO_2_, PvO_2_) and carbon dioxide (PaCO_2_, PvCO_2_), along with the oxygen saturation difference between arterial and peripheral venous blood. A standard analyzer (Radiometer Medical ApS, Brønshøj, Denmark) was utilized to process all blood gas measurements right after collection. Given that the venous blood was drawn from a peripheral source, the resulting venous carbon dioxide and oxygenation metrics were evaluated strictly as peripheral values, avoiding their interpretation as central or mixed venous parameters.

### 2.6. Hemodynamic Measurements

Throughout the surgery, hemodynamic tracking was primarily centered on heart rate (HR) and mean arterial pressure (MAP). The “induction” values refer to the first intraoperative measurements obtained immediately after endotracheal intubation. Initial recordings were taken right after endotracheal intubation. Subsequent measurements were consistently logged at 15 min intervals, specifically at 15, 30, 45, 60, 75, and 90 min post-intubation.

### 2.7. Biomarker Measurement and Laboratory Procedures

To quantify biomarker shifts, venous blood was drawn immediately prior to surgery (T0) and shortly following extubation (T1). Harvested serum was isolated through centrifugation and preserved at −80 °C for subsequent batch processing. The targeted molecules reflected distinct physiological compartments vulnerable to oxygen fluxes: SP-A denoted alveolar epithelial dynamics; sialic acid alongside syndecan-1 mirrored endothelial glycocalyx integrity; IMA served as a proxy for ischemic and oxidative alterations; TNF-α tracked systemic inflammation; and total protein was checked to account for potential fluid-shift hemodilution. All assays relied on commercial enzyme-linked kits (Sigma Diagnostics, St. Louis, MO, USA) executed strictly per the manufacturers’ instructions. Laboratory technicians performed duplicate readings for every specimen while remaining completely blinded to the subjects’ group allocations.

### 2.8. Outcomes

The primary endpoint of this study was the perioperative SP-A response to the allocated oxygenation protocols. The primary comparison was the between-group difference in SP-A change from baseline (T0) to T1, assessed as a group × time interaction. Secondary exploratory biomarker outcomes included sialic acid, total protein, syndecan-1, IMA, and TNF-α. Furthermore, secondary endpoints encompassed lactate levels, arterial and venous blood gas metrics, and intraoperative hemodynamic parameters such as HR and MAP.

### 2.9. Sample Size Calculation

The primary biomarker, surfactant protein-A (SP-A), served as the foundation for our power analysis. Due to the scarcity of literature regarding SP-A dynamics during elective laparoscopic procedures, the required sample size was computed utilizing an anticipated clinically relevant variation in the percentage change of SP-A between the study cohorts. We projected a 27% inter-group difference alongside a shared standard deviation of 40%. Achieving an 80% statistical power with a two-tailed alpha of 0.05 necessitated a minimum of 36 participants per arm. To accommodate potential dropouts or specimen processing failures, the enrollment goal was adjusted to 40 subjects per group, culminating in a total recruitment target of 80 individuals.

### 2.10. Statistical Analysis

All analyses, including sample size estimation, were conducted in R (version 4.5.1; R Foundation for Statistical Computing, Vienna, Austria). Continuous data were summarized with descriptive measures appropriate to their distribution, and categorical variables were reported as counts and percentages. Biomarker distributions were checked before analysis, and within-group as well as between-group comparisons were performed with parametric or nonparametric methods according to distributional characteristics. Baseline continuous variables were compared using either the independent-samples Student’s *t* test or the Mann–Whitney U test, whereas categorical variables were analyzed with the chi-square test. Repeated intraoperative hemodynamic measurements were evaluated using linear mixed-effects models with subject-specific random intercepts. Fixed effects included study group, time, and the group × time interaction. When interaction terms were significant, post hoc pairwise comparisons were performed with Bonferroni adjustment based on estimated marginal means. Effect size estimates are presented as partial eta squared (η^2^p). Hemodynamic findings were treated as secondary descriptive outcomes. No separate baseline-covariate adjustment was applied for induction MAP/HR; instead, the mixed-effects models incorporated the full repeated-measure structure, including the first post-intubation measurement. For the prespecified primary biomarker endpoint, SP-A was analyzed using a repeated-measures framework including group, time, and group × time interaction; the primary inferential comparison was the group × time interaction, representing the between-group difference in perioperative change from T0 to T1. The remaining biomarkers were analyzed as secondary exploratory outcomes. Blood gas variables and lactate were compared between groups. Blood gas variables obtained from peripheral venous samples were handled descriptively and interpreted cautiously because they represented peripheral venous rather than mixed or central venous measurements. All hypothesis tests were two-sided, and *p* < 0.05 was taken to indicate statistical significance. Biomarker analyses were predefined and hypothesis-driven, and formal multiplicity correction was not applied to the primary analytical framework; however, because multiple secondary exploratory biomarker outcomes were examined, unadjusted group × time interaction *p* values are reported together with Holm-adjusted *p* values as a sensitivity analysis for multiplicity. Accordingly, SP-A was treated as the primary confirmatory biomarker analysis, whereas the remaining biomarker analyses were interpreted as exploratory. For clarity of presentation, the main biomarker figures show mean ± SD, exact model-based *p* values are reported in the Results text, and individual paired trajectories for biomarkers with wider dispersion are additionally provided in Supplementary Figures S1 and S2. A modified intention-to-treat sensitivity analysis was not feasible because some randomized patients had non-evaluable primary biomarker data or protocol-deviating ventilation exposure.

## 3. Results

### 3.1. Patient Selection and Study Flow

**Figure 1:**
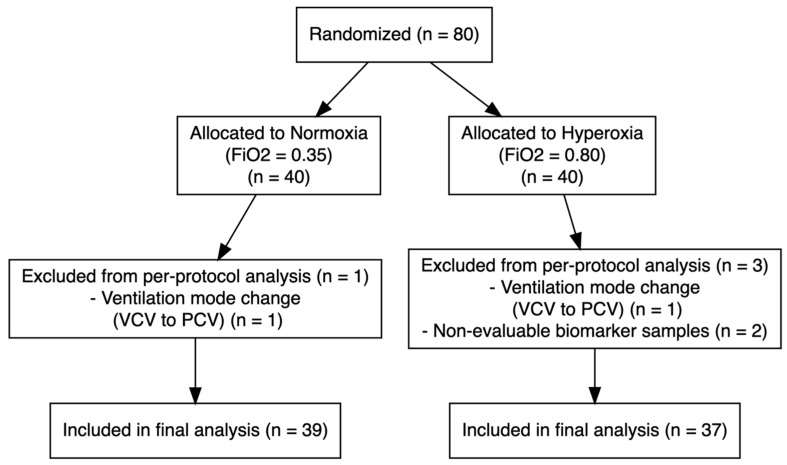
CONSORT flow diagram of patient screening, enrollment, randomization, allocation, and analysis in the normoxia (FiO_2_ 0.35) and hyperoxia (FiO_2_ 0.80) groups.

Eighty individuals meeting the inclusion criteria were randomized equally into hyperoxic (*n* = 40) and normoxic (*n* = 40) groups. Outcome appraisals were strictly based on a per-protocol methodology. Post-randomization, a total of four subjects—three from the hyperoxia arm and one from the normoxia arm—were dropped from the final evaluation matrix. Two patients were excluded because biomarker samples were non-evaluable, and two were excluded because intraoperative ventilation deviated from the protocol-defined strategy. Consequently, the concluding statistical modeling was conducted on a dataset of 76 patients (37 in the hyperoxia cohort and 39 in the normoxia cohort) (Figure 1). Baseline characteristics and exclusion details of the four post-randomization excluded patients are provided in Supplementary File S2.

### 3.2. Patient Characteristics and Perioperative Clinical Data

Baseline demographic and perioperative clinical characteristics of the patients included in the final analysis are presented in Table 1. The two study groups were comparable with respect to age, ASA physical status, height, weight, BMI, duration of surgery, sex distribution, and type of laparoscopic lower abdominal procedure (all *p* > 0.05). The distribution of oncologic versus non-oncologic procedures was similar between groups (29/39 vs. 31/37, *p* = 0.468). Operation duration was also comparable between the normoxia and hyperoxia groups (120.3 ± 39.3 vs. 118.6 ± 32.8 min, *p* = 0.851).

### 3.3. Intraoperative Hemodynamic and Respiratory Changes

Analysis of MAP demonstrated notable fluctuations throughout the monitored duration (*p* < 0.001, η^2^*p* = 0.459), along with distinct temporal trajectories between the two cohorts (interaction of group and time: *p* = 0.004, η^2^p = 0.077). Subsequent post hoc evaluations revealed that post-induction MAP was significantly reduced in the hyperoxic arm; however, no further statistical variances between the groups were observed at subsequent intervals. HR similarly exhibited temporal variations (*p* < 0.001, η^2^p = 0.493) and a significant interaction between group and time (*p* = 0.012, η^2^p = 0.066). Despite this, detailed post hoc assessments yielded no significant HR disparities between the study arms during the rest of the surgical timeframe. Because the “induction” measurement corresponds to the first post-intubation value obtained at the start of the assigned FiO_2_ strategy, before any meaningful oxygen-specific physiological separation would be expected, the lower induction MAP in the hyperoxia group should be interpreted as an early intraoperative imbalance rather than as evidence of an effect of assigned oxygen exposure. Accordingly, the hemodynamic findings remain secondary and descriptive. The exact recorded values for both HR and MAP across all designated assessment points are detailed in Table 2. EtCO_2_ and peak airway pressure both changed significantly over time (both *p* < 0.001), but neither showed a significant group × time interaction (EtCO_2_: *p* = 0.161; peak airway pressure: *p* = 0.871). Accordingly, the available intraoperative respiratory data did not indicate a meaningful between-group difference in ventilatory trajectory during the monitored period.

**Table 2:** Intraoperative hemodynamic and respiratory values at predefined time points and mixed-effects model results.

Parameter	Time Point	Normoxia	Hyperoxia	Time Effect *p*	Group × Time Interaction *p*
MAP (mmHg)	Induction	116.9 ± 12.7	101.5 ± 12.9	**<0.001**	**0.004**
	Intraop 15 min	83.9 ± 9.1	83.2 ± 10.2		
	Intraop 30 min	86.2 ± 8.8	86.3 ± 12.5		
	Intraop 45 min	86.5 ± 9.7	87.8 ± 10.8		
	Intraop 60 min	87.6 ± 9.5	84.5 ± 9.2		
	Intraop 75 min	86.5 ± 8.1	81.0 ± 9.9		
	Intraop 90 min	85.2 ± 11.9	84.1 ± 8.4		
HR (bpm)	Induction	83.6 ± 12.0	77.6 ± 12.2	**<0.001**	**0.012**
	Intraop 15 min	73.6 ± 11.5	72.7 ± 14.5		
	Intraop 30 min	66.6 ± 7.8	70.2 ± 12.1		
	Intraop 45 min	64.4 ± 7.0	65.5 ± 9.0		
	Intraop 60 min	64.1 ± 6.6	64.4 ± 8.4		
	Intraop 75 min	60.6 ± 6.4	64.2 ± 8.7		
	Intraop 90 min	59.9 ± 6.1	64.6 ± 8.9		
EtCO_2_ (mmHg)	Induction	34.9 ± 1.9	35.5 ± 1.8	**<0.001**	0.161
	Intraop 15 min	36.1 ± 1.0	36.1 ± 0.9		
	Intraop 30 min	36.4 ± 0.7	36.9 ± 0.9		
	Intraop 45 min	36.7 ± 1.0	37.3 ± 0.7		
	Intraop 60 min	36.6 ± 1.2	37.0 ± 1.2		
	Intraop 75 min	36.6 ± 1.3	37.3 ± 1.3		
	Intraop 90 min	36.1 ± 1.0	37.0 ± 1.3		
Peak airway pressure (cmH_2_O)	Induction	17.6 ± 1.5	17.2 ± 1.9	**<0.001**	0.871
	Intraop 15 min	21.1 ± 2.3	19.6 ± 3.4		
	Intraop 30 min	22.9 ± 2.3	21.9 ± 3.7		
	Intraop 45 min	23.4 ± 3.2	22.4 ± 4.8		
	Intraop 60 min	23.6 ± 2.8	23.2 ± 3.3		
	Intraop 75 min	23.9 ± 3.4	23.2 ± 3.7		
	Intraop 90 min	22.4 ± 2.8	22.2 ± 3.4		

*MAP, mean arterial pressure; HR, heart rate; EtCO_2_, end-tidal carbon dioxide.*

### 3.4. Perioperative Blood Gas and Metabolic Parameters

Table 3 presents the blood gas metrics recorded ten minutes post-stabilization of the designated oxygen fraction. Consistent with the study design, the hyperoxia group demonstrated substantially higher PaO_2_ compared to the normoxia group (331.5 ± 77.1 vs. 164.0 ± 54.7 mmHg, *p* < 0.001). Additionally, both arterial and peripheral venous oxygen saturation levels were slightly, yet significantly, elevated under hyperoxic conditions. Conversely, no statistically significant differences were observed between the groups regarding pH, PaCO_2_, PvCO_2_, or lactate concentrations. Perioperative hemoglobin concentrations were also comparable between groups. Preoperative hemoglobin levels were 12.82 ± 1.51 g/dL in the normoxia group and 12.23 ± 1.46 g/dL in the hyperoxia group (*p* = 0.236), while postoperative hemoglobin levels were 13.19 ± 1.65 g/dL and 12.30 ± 1.35 g/dL, respectively (*p* = 0.095). In a repeated-measures analysis including group, time, and group × time interaction, the group × time interaction for hemoglobin was not significant (*p* = 0.059; η^2^p = 0.084).

### 3.5. Perioperative Changes in Oxidative Stress, Inflammatory, and Glycocalyx-Related Biomarkers

SP-A was the prespecified primary biomarker endpoint and constitutes the main confirmatory biomarker analysis of the study, whereas syndecan-1, sialic acid, IMA, TNF-α, and total protein were evaluated as secondary exploratory biomarker outcomes.

Measurements of total protein showed no significant differences between the cohorts at either evaluation point. At baseline (T0), the normoxia and hyperoxia groups had concentrations of 6.64 ± 0.50 g/dL and 6.86 ± 0.55 g/dL, respectively (*p* = 0.074). Postoperatively (T1), these values shifted marginally to 6.35 ± 0.50 g/dL and 6.37 ± 0.49 g/dL (*p* = 0.849), demonstrating no major perioperative fluctuations within either arm.

**Figure 2:**
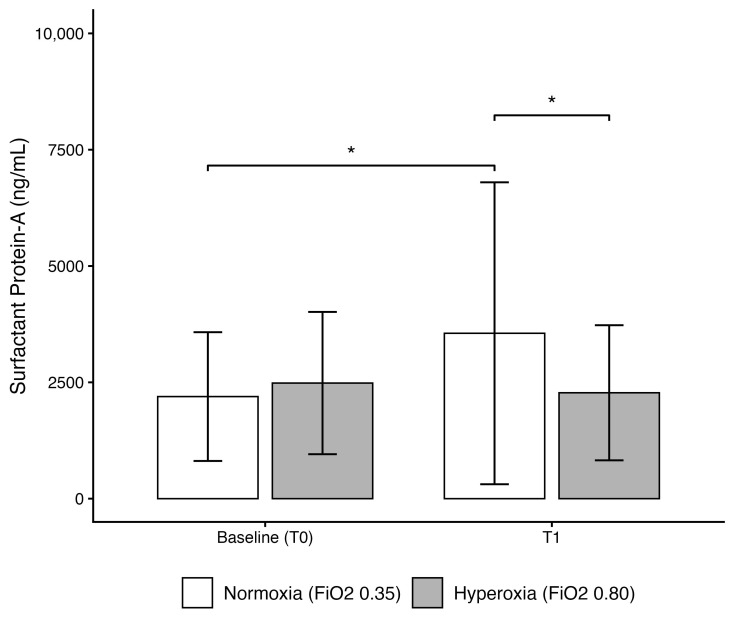
Changes in circulating surfactant protein-A concentrations under normoxic and hyperoxic conditions. Data are presented as mean ± SD; error bars indicate SD. * *p* < 0.05.

SP-A results are shown in Figure 2. Baseline SP-A concentrations were comparable between groups (2194.1 ± 1383.8 ng/mL in the normoxia group vs. 2484.7 ± 1529.2 ng/mL in the hyperoxia group). In a repeated-measures analysis including group, time, and group × time interaction, the group × time interaction was significant (*p* = 0.019; η^2^p = 0.084), indicating that the perioperative SP-A trajectory differed between groups. The estimated between-group difference in change from T0 to T1 (Normoxia minus Hyperoxia) was 1471.5 ng/mL (95% CI 274.9 to 2668.1). This between-group difference in perioperative change constitutes the main inferential result for SP-A. During the perioperative period, SP-A rose in the normoxia group to 3554.6 ± 3244.7 ng/mL at T1 while the hyperoxia group showed no comparable temporal increase (2275.9 ± 1451.9 ng/mL at T1). Postoperative SP-A values were higher in the normoxia group than in the hyperoxia group. As the primary biomarker endpoint, SP-A provides the main inferential basis for the mechanistic interpretation of the study. Individual paired SP-A trajectories are additionally shown in Supplementary Figure S1.

**Figure 3:**
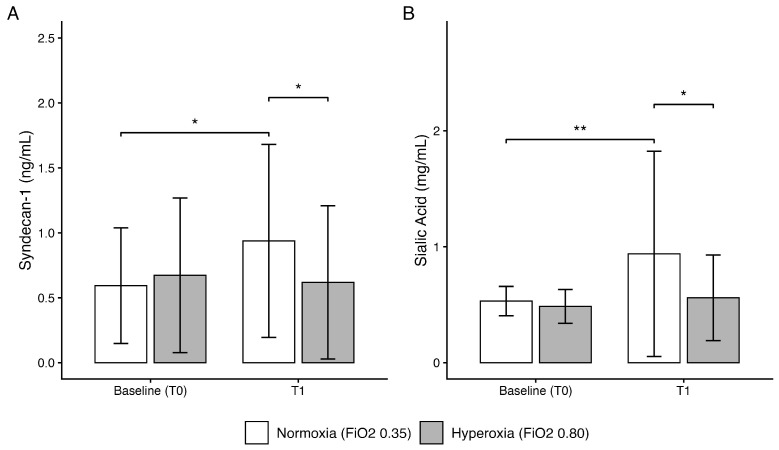
Circulating syndecan-1 (**A**) and sialic acid (**B**) concentrations in patients receiving normoxia (FiO_2_ 0.35) or hyperoxia (FiO_2_ 0.80). Data are presented as mean ± SD; error bars indicate SD. * *p* < 0.05; ** *p* < 0.01.

Figure 3 displays the glycocalyx-related biomarker results. In exploratory repeated-measures analyses, the unadjusted group × time interaction *p* values were 0.130 for syndecan-1 (η^2^*p* = 0.032) and 0.061 for sialic acid (η^2^p = 0.046); after Holm correction, these *p* values were 0.365 and 0.243, respectively. Accordingly, these secondary biomarker findings should be interpreted as exploratory descriptive patterns rather than confirmatory evidence independent of the primary SP-A analysis. Here, too, the relevant inferential comparison is the between-group difference in change over time rather than whether one group reached within-group significance and the other did not. In the normoxia group, syndecan-1 increased from 0.59 ± 0.45 ng/mL at T0 to 0.94 ± 0.74 ng/mL at T1, whereas values in the hyperoxia group remained stable over time (0.67 ± 0.60 ng/mL to 0.62 ± 0.59 ng/mL). A similar descriptive pattern was observed for sialic acid: the normoxia group changed from 0.53 ± 0.13 mg/mL to 0.94 ± 0.89 mg/mL, while the hyperoxia group changed from 0.49 ± 0.15 mg/mL to 0.56 ± 0.37 mg/mL. Individual paired syndecan-1 trajectories are additionally shown in Supplementary Figure S2.

**Figure 4:**
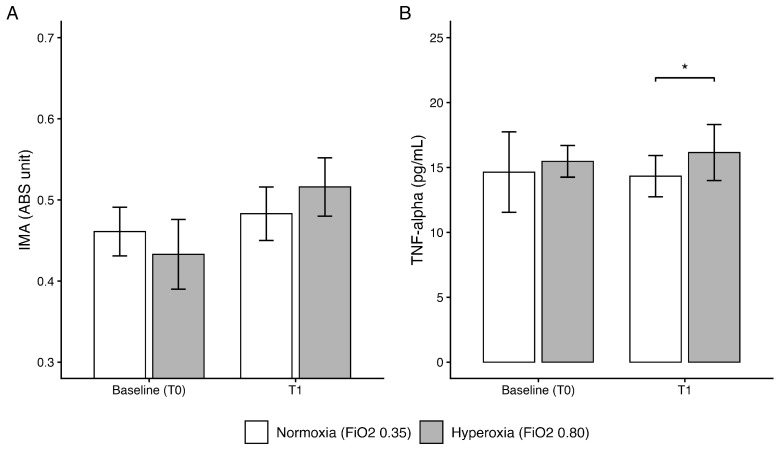
Effects of normoxia and hyperoxia on oxidative stress and inflammatory biomarkers. (**A**) Circulating ischemia-modified albumin (IMA) concentrations in patients receiving normoxia (FiO₂ 0.35) or hyperoxia (FiO₂ 0.80). (**B**) Circulating TNF-α concentrations in patients receiving normoxia (FiO₂ 0.35) or hyperoxia (FiO₂ 0.80). Data are presented as mean ± SD; error bars indicate SD. * *p* < 0.05.

Circulating levels of IMA and TNF-α are shown in Figure 4. In exploratory repeated-measures analyses, the unadjusted group × time interaction *p* values were 0.333 for IMA (η^2^p = 0.012) and 0.122 for TNF-α (η^2^p = 0.036); after Holm correction, both were 0.365. These findings do not support a robust interaction effect for either secondary biomarker after accounting for multiplicity. IMA concentrations were comparable between the normoxia and hyperoxia groups at T0 (0.46 ± 0.03 vs. 0.43 ± 0.04 ABS units). At T1, IMA levels were 0.48 ± 0.03 ABS units in the normoxia group and 0.52 ± 0.04 ABS units in the hyperoxia group, with no statistically significant differences observed between groups or over time. In contrast, TNF-α levels were descriptively higher in the hyperoxia group at T1 (16.2 ± 2.2 pg/mL) compared with the normoxia group (14.3 ± 1.6 pg/mL), whereas baseline TNF-α concentrations were similar between groups (14.6 ± 3.1 vs. 15.5 ± 1.2 pg/mL).

## 4. Discussion

In this prospective randomized study, the two oxygen strategies achieved clear physiological separation, but hyperoxia did not confer a meaningful hemodynamic advantage, as differences in MAP and HR were small and transient. The clearest biomarker pattern emerged in SP-A, while secondary glycocalyx-related biomarkers showed descriptively lower circulating patterns under hyperoxia. By contrast, the inflammatory and oxidative markers did not follow the same directional pattern, with a descriptively higher postoperative TNF-α level under hyperoxia as an isolated exploratory inflammatory signal and no significant between-group difference in IMA. In repeated-measures analyses, a significant group × time interaction was observed for SP-A, whereas no significant group × time interaction was observed for the secondary biomarkers after accounting for multiplicity. Because SP-A was the prespecified primary biomarker endpoint, the main confirmatory interpretation of the study is centered on the SP-A findings, whereas the remaining biomarker results should be regarded as secondary exploratory observations. Accordingly, in the context of this randomized comparison, the primary inferential emphasis should be placed on the between-group difference in perioperative change rather than on within-group significance considered separately. Overall, these findings suggest that perioperative hyperoxia was associated with a distinct pattern of circulating biomarker modulation across pulmonary epithelial, glycocalyx-related, inflammatory, and oxidative domains, rather than direct evidence of reduced tissue injury or a uniform biological effect across all measured domains. Taken together, this mixed biomarker profile supports a domain-specific and biomarker-specific response to perioperative oxygen exposure rather than a unified mechanistic effect.

With respect to hemodynamics, our findings suggest that hyperoxia did not confer a meaningful circulatory advantage. Although the temporal profiles of MAP and HR differed between groups, these differences were limited and did not translate into sustained between-group separation. This is broadly consistent with previous evidence indicating that increased arterial oxygen tension does not necessarily result in clinically relevant macrocirculatory benefit [1,2,6,7]. In this context, the hemodynamic effects observed in our cohort appear modest relative to the more distinct biological effects seen across biomarker domains. Likewise, the alveolar–arterial oxygen gradient and PaO_2_/FiO_2_ ratio were interpreted only as descriptive physiological indicators within the context of the assigned FiO_2_ strategy, rather than as standalone evidence of pathological gas exchange impairment.

The clinical implications of perioperative hyperoxia remain controversial. In our study, hyperoxia did not confer a clear hemodynamic advantage, but neither did the biomarker profile support a uniformly adverse interpretation. Instead, the most prominent finding under hyperoxia was a different circulating biomarker pattern involving SP-A and glycocalyx-related markers, whereas the inflammatory signal was limited to TNF-α. This distinction is important in light of previous studies suggesting that higher perioperative FiO_2_ may provide benefit in selected settings, particularly with respect to SSI or attenuation of postoperative inflammatory responses. For example, Schietroma et al. reported lower postoperative inflammatory markers and less postoperative immunosuppression with 80% oxygen in laparoscopic Nissen fundoplication, while other studies suggested possible benefit for superficial SSI or abdominal surgery-related SSI under selected conditions [10,18,19,20]. However, larger randomized and observational studies have not shown a consistent clinical benefit and have raised concern regarding pulmonary complications and longer-term adverse outcomes [3,4,7,21,22,23]. Taken together with our findings, the current evidence suggests that perioperative hyperoxia should not be viewed as uniformly beneficial or uniformly harmful; rather, its biological and clinical effects may vary across specific domains, outcomes, and time points.

One of the most notable findings of our study was the different SP-A trajectory observed under hyperoxia. Circulating SP-A increased significantly in the normoxia group, whereas no comparable increase was observed under hyperoxic conditions. This descriptive pattern was supported by the repeated-measures analysis, which demonstrated a significant group × time interaction for SP-A, indicating that the perioperative SP-A trajectory differed between the two oxygen strategies. As the prespecified primary biomarker endpoint, SP-A was interpreted on the basis of both this repeated-measures result and the descriptive perioperative pattern. Given that SP-A is a major surfactant-associated glycoprotein and a widely used marker of alveolar epithelial stress and surfactant-related activity, this finding should not be interpreted as direct evidence of reduced pulmonary epithelial injury under hyperoxia. Rather, SP-A may reflect a more complex biological response involving surfactant homeostasis, innate immune defense, and alveolar surface interactions [11,24]. Consistent with this cautious interpretation, the available intraoperative respiratory parameters, namely EtCO_2_ and peak airway pressure, did not show a significant group × time interaction, arguing against a major difference in the monitored ventilatory trajectory between groups during the study period. In addition, recent evidence indicates that SP-A may bind strongly to sulfated glycosaminoglycans within the alveolar epithelial glycocalyx, supporting surfactant stability and function and suggesting that changes in circulating SP-A may reflect differential regulation, binding, or release rather than a simple linear injury signal [25]. This interpretation should be considered in the context of a broader literature in which the pulmonary effects of higher inspired oxygen fractions remain mixed: experimental and translational studies have suggested that supraphysiological oxygen exposure can influence epithelial and surfactant biology, whereas clinical perioperative data have not consistently demonstrated a uniformly harmful molecular or pulmonary profile [1,6,21,22,23]. In addition, hyperoxia may promote absorption atelectasis and related regional mechanical stress, and this potential mechanism cannot be excluded as a contributor to the observed SP-A response in the present study. Within this framework, our findings raise the possibility that, in the present laparoscopic setting, hyperoxia was associated with a lower circulating SP-A response and a distinct pattern of SP-A-related biological regulation, rather than as direct evidence of reduced pulmonary injury. However, in the absence of additional lung-specific biomarkers, this interpretation should be regarded as hypothesis-generating rather than definitive evidence of either reduced injury or a specific protective pulmonary effect.

Beyond SP-A, the glycocalyx-related markers showed a descriptively different circulating pattern under hyperoxia. Syndecan-1 and sialic acid increased under normoxia rather than hyperoxia. However, because these biomarkers were analyzed as secondary exploratory outcomes and their interaction findings did not remain significant after Holm correction, they should be interpreted cautiously and as supportive rather than confirmatory observations. Given that syndecan-1 is a widely used marker of glycocalyx shedding and endothelial surface injury, and that sialic acid may also reflect disruption of the endothelial surface layer, the lower circulating pattern observed under hyperoxia should not be interpreted as direct evidence of reduced endothelial injury. Rather, these findings may indicate a different glycocalyx-related biomarker response in the perioperative period [12,13,26,27]. This interpretation should nevertheless be made with caution, because circulating glycocalyx-related markers may be influenced not only by the extent of endothelial injury itself, but also by timing of release, vascular compartment, mechanical ventilation, pneumoperitoneum, perioperative hemodynamics, and other aspects of surgical stress. In this context, our findings suggest that perioperative hyperoxia was associated with a lower early glycocalyx-related biomarker response rather than a simple amplification of endothelial injury. This interpretation is also compatible with evidence that oxygen-related vascular effects are heterogeneous across biomarkers and vascular beds; in the ROCS randomized clinical trial, for example, intraoperative hyperoxia impaired endothelium-independent vasodilation and increased selected oxidative and vascular stress markers, yet did not significantly alter circulating syndecan-1 concentrations [14,26]. Taken together, these observations suggest that glycocalyx biology under perioperative oxygen exposure is complex and context-dependent, and that lower circulating syndecan-1 and sialic acid levels under hyperoxia should be interpreted as a lower biomarker response rather than as direct proof of reduced endothelial injury.

The inflammatory findings in our study should be interpreted with greater caution. TNF-α concentrations were higher in the hyperoxia group at the end of surgery. TNF-α is a key pro-inflammatory cytokine involved in endothelial activation, oxidative injury amplification, and perioperative inflammatory signaling. Experimental work supports the plausibility of hyperoxia-related inflammatory activation, with animal and cellular studies demonstrating increased cytokine release, including TNF-α, IL-1β, and IL-6, under severe or prolonged hyperoxic exposure [9,28,29,30]. By contrast, human perioperative data are less uniform, and some studies have reported attenuated postoperative inflammatory markers under higher FiO_2_ in specific surgical settings, whereas broader perioperative reviews suggest that the biological effects of hyperoxia may depend on exposure duration, tissue vulnerability, and the balance between oxidative and antioxidant responses [1,2,6,8,10]. Importantly, TNF-α was the only inflammatory marker assessed in the present study and therefore cannot, by itself, characterize the overall inflammatory cascade. This is particularly relevant in the perioperative setting, where inflammatory mediators are temporally dynamic and may peak at different postoperative intervals, while short-term hyperoxia has not consistently produced a clear systemic cytokine response in humans [31,32]. In addition, TNF-α was analyzed as a secondary exploratory biomarker, and its interaction finding was not robust after Holm correction. Accordingly, the descriptively higher TNF-α level observed in our study should be interpreted as an isolated inflammatory signal within a biologically heterogeneous profile rather than as evidence of a generalized pro-inflammatory effect of hyperoxia.

In contrast, the absence of a significant between-group difference in IMA suggests that not all oxidative stress-related pathways were equally affected by perioperative oxygen exposure. IMA is known to be context-dependent, and its sensitivity may be limited in short perioperative conditions [16,33]. Prior perioperative studies have also suggested that higher FiO_2_ may preferentially influence some oxidative pathways more than others [8,34,35,36,37]. Therefore, the lack of change in IMA in our study likely reflects the heterogeneous and marker-specific nature of oxidative stress responses rather than the absence of a biological effect. Considered together, the TNF-α and IMA findings reinforce the view that perioperative oxygen exposure may influence different biological domains in a biomarker-specific manner rather than through a single uniform mechanism.

From a clinical perspective, our findings do not support a clear hemodynamic advantage of liberal oxygen administration during elective laparoscopic surgery. In non-hypoxemic patients, higher FiO_2_ was not associated with circulatory benefit, while the accompanying biomarker profile was characterized mainly by a lower circulating SP-A pattern and descriptively lower glycocalyx-related biomarker responses, rather than by a uniformly adverse pattern. The isolated increase in TNF-α should be interpreted cautiously and does not, by itself, outweigh the broader pulmonary epithelial and glycocalyx-related biomarker signal observed under hyperoxia. Taken together, these findings suggest that perioperative oxygen should not be regarded as a purely neutral supportive intervention, but rather as a biologically active exposure whose effects may vary across domains and outcomes. These biomarker findings should not be extrapolated to support that FiO_2_ 0.80 is clinically safer, protective, or preferable in perioperative practice. However, given the mechanistic nature of the present study, these observations require confirmation in larger outcome-driven trials [1,2,6,26,27,38]. Because no pulmonary complications, postoperative oxygenation outcomes, atelectasis assessments, endothelial function measurements, recovery variables, or surgical site infection outcomes were collected, the translational significance of the observed biomarker changes remains uncertain. These early biomarker findings therefore cannot be directly linked to clinically relevant postoperative events.

### Strengths and Limitations

This study has several strengths. First, its prospective randomized design and the clear physiological separation between the two oxygen strategies strengthen interpretation of the observed biological differences. Second, the use of a multidomain biomarker approach allowed simultaneous assessment of pulmonary epithelial, endothelial glycocalyx-related, inflammatory, and oxidative stress pathways, providing a more integrated characterization of perioperative oxygen effects than studies focusing on a single biological domain. This design may help capture complex and potentially heterogeneous biological responses that are not readily detected in conventional outcome-driven studies. However, some limitations should be considered. The study was conducted at a single center, and the sample size was designed for mechanistic evaluation rather than clinical outcomes. Biomarker measurements were limited to baseline and a single early postoperative time point shortly after extubation. Therefore, delayed, biphasic, or biomarker-specific postoperative responses may have been missed, particularly for dynamic inflammatory, oxidative, endothelial, and surfactant-related markers. Additional postoperative sampling at later time points, such as 2 h, 6 h, 24 h, or postoperative day 1, would have provided a more complete temporal characterization of these biological responses. Although total protein and perioperative hemoglobin were available as indirect markers of dilution, detailed perioperative hematocrit, crystalloid/colloid administration, urine output, blood loss, and net fluid balance data were not systematically available. Therefore, the potential contribution of hemodilution, intravascular volume changes, and redistribution to the observed circulating biomarker concentrations cannot be fully excluded. Another limitation is the wide dispersion of some circulating biomarkers, particularly SP-A and syndecan-1, which warrants cautious interpretation of between-group differences. For this reason, the main summary figures were complemented by supplementary paired trajectory plots to better display within-subject variability. Direct perioperative assessment of atelectasis or lung impairment, such as lung ultrasound or imaging, was not performed, and postoperative pulmonary complication or surgical site infection data were not collected. Therefore, the pulmonary biomarker findings could not be directly linked to structural lung changes or clinically relevant postoperative outcomes. Detailed ventilatory mechanics and pulmonary physiology variables—such as plateau pressure, driving pressure, respiratory system compliance, recruitment maneuvers, postoperative oxygenation, and atelectasis assessment—were not systematically available. Although intraoperative EtCO_2_ and peak airway pressure data were available and showed no significant group × time interaction, these surrogate parameters do not fully exclude the possible contribution of ventilation mechanics and pneumoperitoneum-related respiratory effects to the observed SP-A pattern. Accordingly, these biomarker patterns should be interpreted as exploratory and hypothesis-generating rather than outcome-based clinical evidence. Because multiple biomarkers and repeated comparisons were analyzed without formal multiplicity correction, the risk of type I error cannot be excluded and significant findings should be interpreted cautiously. In addition, some of the selected biomarkers are not entirely specific to a single biological pathway, which may limit interpretation of the findings. The per-protocol analysis may also have introduced bias. Although the number of excluded randomized patients was small, per-protocol analysis may still increase the risk of selection bias and may overestimate treatment effects relative to an intention-to-treat approach. A formal intention-to-treat sensitivity analysis was not feasible because primary biomarker data were unavailable in participants with non-evaluable samples, and ventilation protocol deviations affected the intended exposure conditions. Furthermore, despite prospective conduct and ethics approval before enrollment, retrospective trial registration remains a methodological limitation because it reduces confidence in the complete prespecification of outcomes and analyses. Accordingly, the study should not be interpreted as providing strong confirmatory evidence, and its findings are more appropriately viewed within an exploratory mechanistic framework. In addition, the study population included both oncologic and non-oncologic procedures. Although the distribution of procedure type and operation duration was similar between groups, the study was underpowered for robust subgroup-adjusted inference, particularly given the predominance of oncologic procedures and the limited non-oncologic subgroup. Finally, the study does not provide information on whether the observed biological changes are associated with clinically meaningful outcomes. Accordingly, the present findings should not be used to infer that hyperoxia is clinically safer, protective, or preferable.

## 5. Conclusions

In conclusion, perioperative oxygen concentration was associated with distinct early biological responses in elective laparoscopic surgery. Hyperoxia did not provide a clear hemodynamic advantage, but was associated with different early circulating SP-A and glycocalyx-related biomarker trajectories in the early perioperative period. These findings should be interpreted as a pattern of differential biomarker modulation rather than as direct evidence of reduced tissue injury or a specific protective effect of hyperoxia. They should not be interpreted as evidence of pulmonary epithelial or endothelial protection. They also cannot be directly linked to postoperative pulmonary complications, atelectasis, endothelial dysfunction, surgical site infection, or other clinical outcomes. Accordingly, FiO_2_ 0.80 should not be regarded as clinically safer, protective, or preferable on the basis of the present data. The higher TNF-α level observed under hyperoxia should be interpreted cautiously, as it represented an isolated inflammatory signal. Overall, these findings indicate that FiO_2_ 0.80 was associated with a different early biomarker pattern, the clinical meaning of which remains unknown. Because SP-A was the prespecified primary biomarker endpoint, the strongest inferential weight of the study rests on the SP-A analysis, whereas the non-primary biomarker findings should be interpreted as secondary exploratory observations. Given the mechanistic nature of the study and the absence of formal multiplicity correction, these observations should be regarded as exploratory and hypothesis-generating. They should not be interpreted as confirmatory evidence of a prespecified causal effect of hyperoxia on pulmonary epithelial or glycocalyx-related injury. Further studies integrating mechanistic biomarkers with clinically relevant outcomes are needed to define the optimal oxygen strategy in surgical patients.

## Figures and Tables

**Table 1:** Baseline demographic and clinical characteristics of the study groups.

Variable	Normoxia Group (FiO_2_ 0.35, *n* = 39)	Hyperoxia Group (FiO_2_ 0.80, *n* = 37)	*p*
***Age*** ***(years)***	47.9 ± 12.5	48.9 ± 10.9	0.708
***ASA physical status***	1.7 ± 0.5	1.8 ± 0.5	0.736
***Height (cm)***	168.2 ± 7.5	166.1 ± 7.9	0.243
***Weight (kg)***	75.4 ± 9.4	75.7 ± 11.6	0.916
***BMI (kg/m^2^)***	26.7 ± 3.1	27.4 ± 3.6	0.353
***Duration of surgery. (min)***	124.0 ± 45.6	123.5 ± 35.4	0.962
***Sex (male/female, %)***	40/60	34/66	0.603
***Type of laparoscopic procedure, n (%)***			0.494
*Hernia repair*	5 (12.8)	4 (10.8)	
*Oncologic procedures*	29 (74.4)	31 (83.8)	
*Appendectomy*	5 (12.8)	2 (5.4)	

*FiO_2_, fraction of inspired oxygen; ASA, American Society of Anesthesiologists; BMI, body mass index; SD, standard deviation.*

**Table 3:** Blood gas parameters measured 10 min after establishment of the assigned oxygen fraction.

Variable	Normoxia	Hyperoxia	*p*
***Arterial*** ***pH***	7.42 ± 0.06	7.41 ± 0.02	0.796
***PaO_2_ (mmHg)***	164.0 ± 54.7	331.5 ± 77.1	**<0.001**
***PaCO_2_ (mmHg)***	38.9 ± 3.6	37.9 ± 3.0	0.186
***SaO_2_ (%)***	98.9 ± 0.8	99.8 ± 0.4	**<0.001**
***Venous pH***	7.40 ± 0.06	7.40 ± 0.02	0.881
***PvO_2_ (mmHg)***	92.0 ± 31.1	185.0 ± 66.2	**<0.001**
***PvCO_2_ (mmHg)***	41.4 ± 3.4	40.5 ± 2.8	0.210
**Peripheral venous oxygen saturation** ***(%)***	95.3 ± 4.3	98.6 ± 1.2	**<0.001**
**Arterial–peripheral venous O_2_ saturation difference** ***(%)***	3.7 ± 4.1	1.2 ± 1.2	**<0.001**
***Lactate (mmol/L)***	1.08 ± 0.50	1.02 ± 0.30	0.498
***The alveolar–arterial oxygen gradient***	24.0 ± 62.4	200.8 ± 86.7	**<0.001**
***PaO_2_/FiO_2_ ratio***	505.3 ± 180.5	402.9 ± 108.8	0.077

*PaO_2_, arterial partial pressure of oxygen; PaCO_2_, arterial partial pressure of carbon dioxide; SaO_2_, arterial oxygen saturation; PvO_2_, venous partial pressure of oxygen; PvCO_2_, venous partial pressure of carbon dioxide, FiO_2_, fraction of inspired oxygen.*

## Data Availability

To protect patient privacy and confidentiality, the primary data underlying this study are not publicly shared. However, anonymized data subsets can be made available by the corresponding author upon reasonable inquiry, pending adherence to institutional data protection regulations.

## Supplementary Materials

The following supporting information can be downloaded at: https://www.mdpi.com/article/10.3390/life16071160/s1, Supplementary Figure S1: Individual paired SP-A trajectories; Supplementary Figure S2: Individual paired Syndecan-1 trajectories; Supplementary File S1: chronology, endpoint prespecification, and document-consistency details; Supplementary File S2: baseline characteristics and exclusion details of the four post-randomization excluded patients.

## Abbreviations

ASA, American Society of Anesthesiologists; BMI, Body Mass Index; CONSORT, Consolidated Standards of Reporting Trials; EtCO_2_, End-Tidal Carbon Dioxide; FiO_2_, Fraction of Inspired Oxygen; HR, Heart Rate; IMA, Ischemia-Modified Albumin; MAP, Mean Arterial Pressure; PaCO_2_, Arterial Partial Pressure of Carbon Dioxide; PaO_2_, Arterial Partial Pressure of Oxygen; PCV, Pressure-Controlled Ventilation; PEEP, Positive End-Expiratory Pressure; PvCO_2_, Venous Partial Pressure of Carbon Dioxide; PvO_2_, Venous Partial Pressure of Oxygen; ROS, Reactive Oxygen Species; SaO_2_, Arterial Oxygen Saturation; SD, Standard Deviation; SP-A, Surfactant Protein-A; SSI, Surgical Site Infection; T0, Baseline Time Point; T1, End-of-Surgery/Postextubation Time Point; TNF-α, Tumor Necrosis Factor-Alpha; VCV, Volume-Controlled Ventilation.
