# Supplementary material for: Perioperative Hyperoxia and Early Pulmonary Epithelial and Glycocalyx-Related Biomarker Trajectories in Laparoscopic Surgery: A Prospective Randomized Study
Source: Life (Basel). 2026 Jul 14;16(7):1160. doi: 10.3390/life16071160 (PMC13413075; doi:10.3390/life16071160)
Supplement: Supplementary file 1 [file life-16-01160-s001.zip › Supplementary File S1.pdf]

Supplementary File S1. Trial Chronology, Endpoint Prespecification, and Consistency Across Study Documents

Trial Chronology

Ethics approval for the study was obtained on 11 July 2017. The first patient was enrolled on 21 September 2017, and the final patient was enrolled on 30 January 2018. An earlier version of the study was presented at the Turkish Society of Anesthesiology and Reanimation Congress (TARK), held on 7–10 November 2019. The study was retrospectively registered at ClinicalTrials.gov on 10 April 2026.

Endpoint Prespecification

The study was conducted under prior ethics approval and according to the approved protocol. Based on the ethics-approved study framework and the registry record, surfactant protein-A (SP-A) was prespecified as the primary biomarker endpoint before data analysis. In the current manuscript, syndecan-1, sialic acid, ischemia-modified albumin (IMA), tumor necrosis factor-alpha (TNF-α), and total protein are presented as secondary exploratory biomarker outcomes.

Consistency Across Study Documents

The ethics-approved protocol and the retrospective ClinicalTrials.gov record were aligned with respect to the core study framework, including the randomized comparison of FiO_2 0.35 versus FiO_2 0.80, the perioperative biomarker sampling design, and the overall mechanistic focus of the study. However, the prior congress abstract represented an earlier conference-stage summary of the study and placed greater emphasis on glycocalyx-related biomarkers, including a syndecan-based sample size statement. The congress presentation therefore differed in emphasis from the current full manuscript.

The current manuscript should be understood as the full journal report of the study, with a clearer endpoint hierarchy and a more explicit analytical structure. In this version, SP-A is identified as the prespecified primary biomarker endpoint, while the remaining biomarkers are

treated as secondary exploratory outcomes. The current manuscript also provides a more detailed statistical presentation, including repeated-measures analyses and a more cautious interpretation of the non-primary biomarker findings.

Interpretive Implication

Because trial registration was completed after patient enrollment had begun and after enrollment had been completed, the study should be interpreted within an exploratory mechanistic framework rather than as strong confirmatory evidence. The retrospective timing of registration reduces confidence in the complete prospective documentation of endpoint hierarchy and analysis details, even though the study itself was conducted under prior ethics approval.
