# Supplementary material for: Perioperative Hyperoxia and Early Pulmonary Epithelial and Glycocalyx-Related Biomarker Trajectories in Laparoscopic Surgery: A Prospective Randomized Study
Source: Life (Basel). 2026 Jul 14;16(7):1160. doi: 10.3390/life16071160 (PMC13413075; doi:10.3390/life16071160)
Supplement: Supplementary file 1 [file life-16-01160-s001.zip › Supplementary File S2.pdf]

Supplementary File S2. Baseline Characteristics and Exclusion Details of Post-Randomization Excluded Patients

**Excluded patient**	**Randomized group**	**Age (years)**	**Sex**	**ASA class**	**Procedure type**	**Reason for exclusion**	**Timing of exclusion determination**	**Primary biomarker data availability**
Patient 1	Normoxia	70	Female	II	Hernia repair	Intraoperative ventilation mode change (VCV to PCV)	Determined intraoperatively, before per-protocol biomarker evaluation	Not included in final biomarker analysis
Patient 2	Hyperoxia	62	Male	II	Hernia repair	Intraoperative ventilation mode change (VCV to PCV)	Determined intraoperatively, before per-protocol biomarker evaluation	Not included in final biomarker analysis
Patient 3	Hyperoxia	51	Male	I	Appendectomy	Non-evaluable biomarker sample due to hemolysis	Determined after sampling but before any valid biomarker result was available	Sample obtained but non-evaluable
Patient 4	Hyperoxia	53	Male	I	Hernia repair	Non-evaluable biomarker sample due to hemolysis	Determined after sampling but before any valid biomarker result was available	Sample obtained but non-evaluable

* No formal statistical comparison was performed for excluded patients because of the very small number of post-randomization exclusions (n = 4). This table is provided to improve transparency regarding the per-protocol analytic population. In the hemolyzed-sample cases, exclusion was based on sample non-evaluability rather than on observed biomarker findings.
