# Supplementary figures and images for: Perioperative Hyperoxia and Early Pulmonary Epithelial and Glycocalyx-Related Biomarker Trajectories in Laparoscopic Surgery: A Prospective Randomized Study
Source: Life (Basel). 2026 Jul 14;16(7):1160. doi: 10.3390/life16071160 (PMC13413075; doi:10.3390/life16071160)

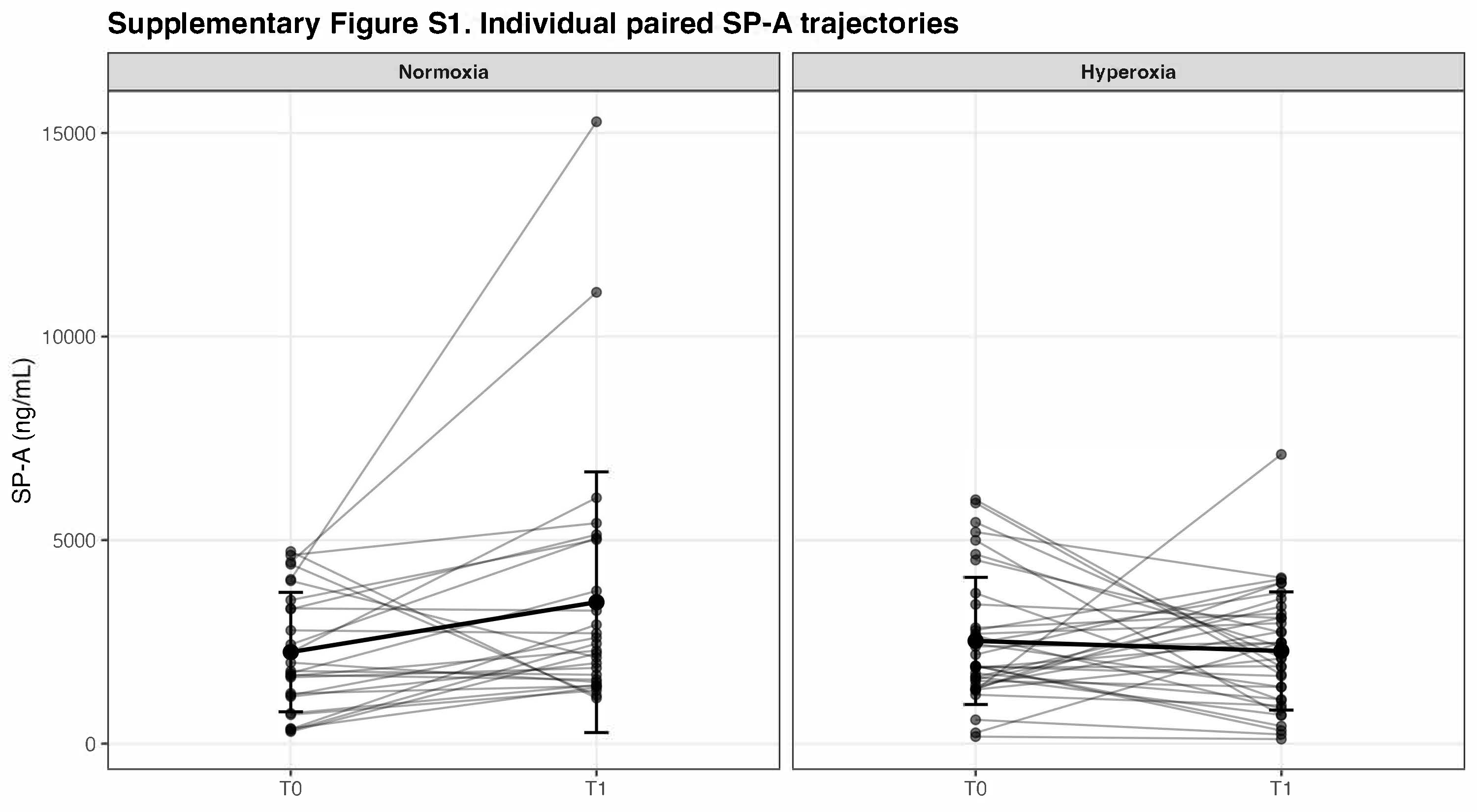

Supplement: Supplementary file 1 [file life-16-01160-s001.zip › Supplementary_Figure_S1.png]

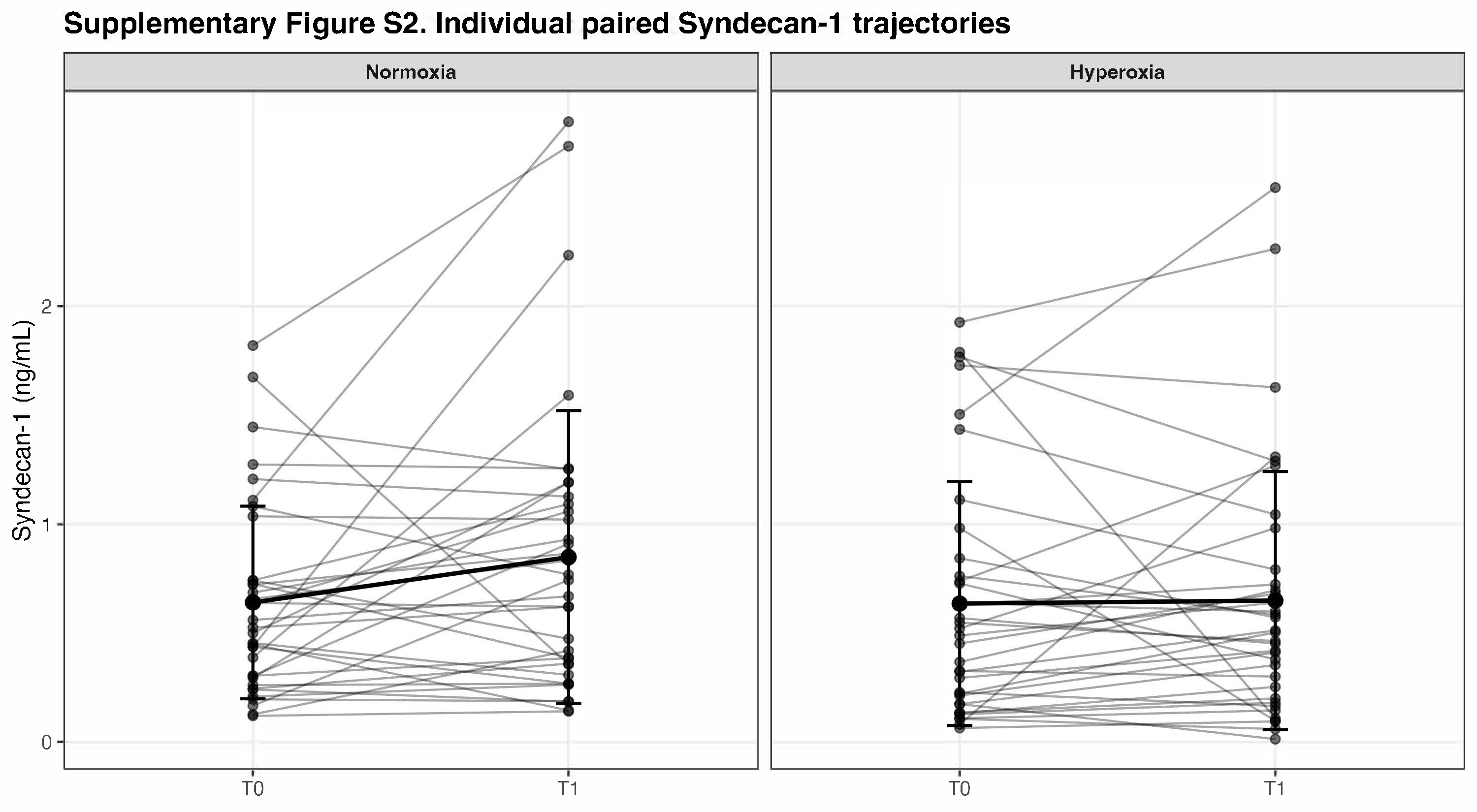

Supplement: Supplementary file 1 [file life-16-01160-s001.zip › Supplementary_Figure_S2.png]
